# Robotic right lower lobe sleeve lobectomy using the single-port robotic system: Challenging case after immunotherapy

**DOI:** 10.1016/j.xjtc.2025.09.004

**Published:** 2025-09-17

**Authors:** Byung Mo Gu, Jun Hee Lee, Hyun Koo Kim

**Affiliations:** Department of Thoracic and Cardiovascular Surgery, Korea University Guro Hospital, Korea University College of Medicine, Seoul, Republic of Korea

**Figure:**
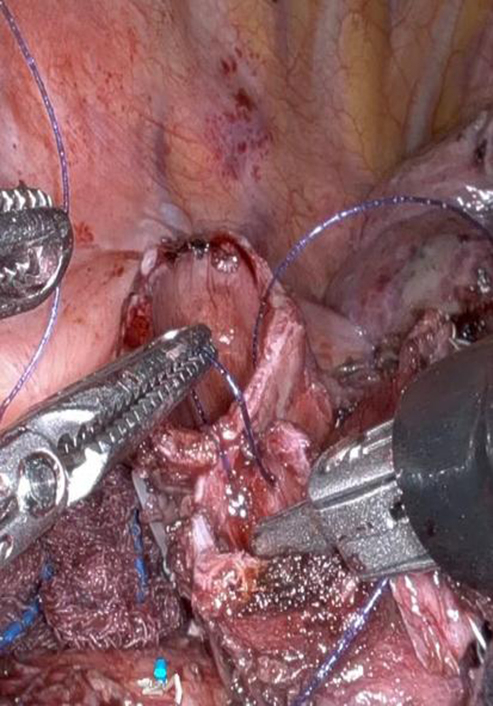
Bronchial anastomosis with barbed sutures under controlled traction using robotic arms.

**Central Message**

The single-port robotic system facilitates safe and reliable performance of complex lobectomies, even after neoadjuvant chemoimmunotherapy.

A 55-year-old man with squamous cell carcinoma of the right lower lobe presented with a central mass invading the bronchus (cT3N2). After 3 cycles of neoadjuvant chemoimmunotherapy (nivolumab, paclitaxel, carboplatin), restaging positron emission tomography-computed tomography 2 weeks later showed a partial response. Surgery was performed 2 weeks thereafter. The operation was performed using the da Vinci single-port (SP) system (Intuitive Surgical). The tumor was located at the orifice of the superior segmental bronchus (B6) with limited extension into the bronchus intermedius. Intraoperative frozen section confirmed no invasion of the middle lobe bronchus, and sleeve resection was performed preserving the right middle lobe. The middle lobe remained well expanded postoperatively without air leak. Pathology demonstrated major response of the primary lesion with single nodal metastasis (ypT0N1). The patient subsequently received adjuvant paclitaxel-carboplatin chemotherapy.

## Surgical Procedure

The patient was placed in the left lateral decubitus position, and a 4-cm subcostal incision was made along the anterior margins of the seventh to ninth ribs. The diaphragm was incised at its lateral attachment under thoracoscopic guidance. Three traction sutures were used to retract the diaphragm. The robotic arms were introduced through the SP access port. Two Cadière forceps in arm 1 and 2 provided tissue grasping and traction, whereas a Maryland bipolar forceps in arm 3 (right hand) was used for precise dissection. Bronchial anastomosis was performed with absorbable barbed sutures using a robotic needle driver, and a submersion test confirmed airtight closure. The subcostal tunnel was closed by reapproximating both the abdominal musculature and retracted diaphragm in a single layer to prevent herniation (see Video 1).

## Conclusions

The SP system enables not only standard^1^ but also complex lobectomies to be performed safely and reliably, even after neoadjuvant chemoimmunotherapy. Barbed sutures may enhance the efficiency and precision of bronchial anastomosis. Further case studies are required to confirm the overall feasibility and reproducibility of this approach. Institutional review board approval was waived for this single case video, and written informed consent for publication was obtained from the patient.

## Conflict of Interest Statement

The authors reported no conflicts of interest.

The *Journal* policy requires editors and reviewers to disclose conflicts of interest and to decline handling or reviewing manuscripts for which they may have a conflict of interest. The editors and reviewers of this article have no conflicts of interest.

## Supplementary Data

Video 1 Demonstration of a robotic right lower lobe sleeve lobectomy using the single-port robotic system after chemoimmunotherapy, including dissection, bronchial sleeve resection, and anastomosis. Video available at: https://www.jtcvs.org/article/S2666-2507(25)00368-2/fulltext.
