# Supplementary figures and images for: Robotic right lower lobe sleeve lobectomy using the single-port robotic system: Challenging case after immunotherapy
Source: JTCVS Tech. 2025 Sep 17;34:235–6. doi: 10.1016/j.xjtc.2025.09.004 (PMC12683025; doi:10.1016/j.xjtc.2025.09.004)

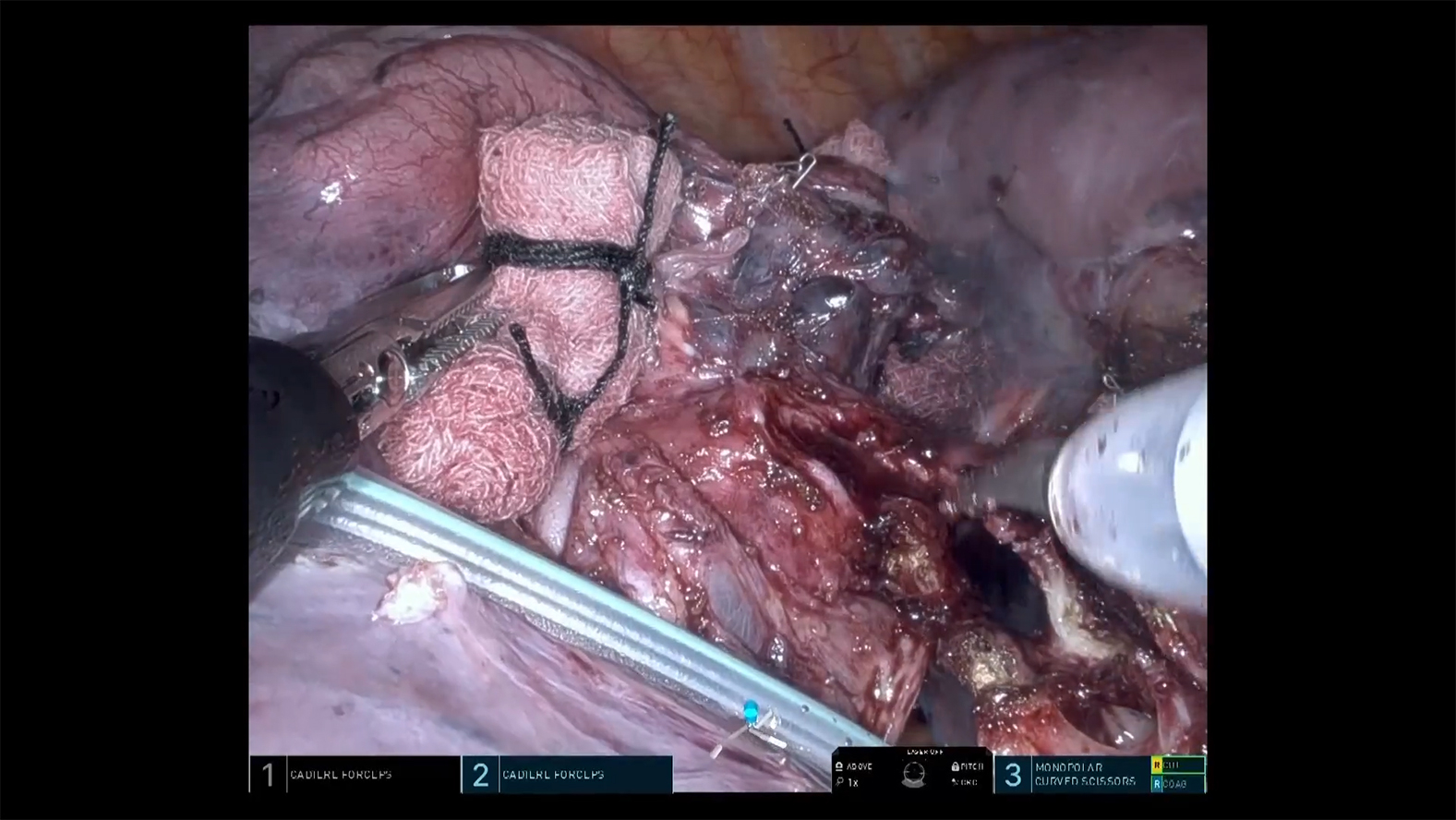

Supplement: Video 1 — Demonstration of a robotic right lower lobe sleeve lobectomy using the single-port robotic system after chemoimmunotherapy, including dissection, bronchial sleeve resection, and anastomosis. Video available at: https://www.jtcvs.org/article/S2666-2507(25)00368-2/fulltext. [file fx2.jpg]
